# Electrostimulation to reduce synaptic scaling driven progression of Alzheimer's disease

**DOI:** 10.3389/fncom.2014.00039

**Published:** 2014-04-03

**Authors:** Mark S. Rowan, Samuel A. Neymotin, William W. Lytton

**Affiliations:** ^1^School of Computer Science, University of BirminghamBirmingham, UK; ^2^Department Physiology and Pharmacology, State University of New York Downstate Medical CenterBrooklyn, NY, USA; ^3^Department Neurobiology, Yale University School of MedicineNew Haven, CT, USA; ^4^Department Neurology, State University of New York Downstate Medical CenterBrooklyn, NY, USA; ^5^Department Neurology, Kings County Hospital CenterBrooklyn, NY, USA

**Keywords:** computer modeling, Alzheimer's disease, homeostasis, synaptic scaling, neocortex, information transfer, neuronal networks, electrostimulation

## Abstract

Cell death and synapse dysfunction are two likely causes of cognitive decline in AD. As cells die and synapses lose their drive, remaining cells suffer an initial decrease in activity. Neuronal homeostatic synaptic scaling then provides a feedback mechanism to restore activity. This homeostatic mechanism is believed to sense levels of activity-dependent cytosolic calcium within the cell and to adjust neuronal firing activity by increasing the density of AMPA synapses at remaining synapses to achieve balance. The scaling mechanism increases the firing rates of remaining cells in the network to compensate for decreases in network activity. However, this effect can itself become a pathology, as it produces increased imbalance between excitatory and inhibitory circuits, leading to greater susceptibility to further cell loss via calcium-mediated excitotoxicity. Here, we present a mechanistic explanation of how directed brain stimulation might be expected to slow AD progression based on computational simulations in a 470-neuron biomimetic model of a neocortical column. The simulations demonstrate that the addition of low-intensity electrostimulation (neuroprosthesis) to a network undergoing AD-like cell death can raise global activity and break this homeostatic-excitotoxic cascade. The increase in activity within the remaining cells in the column results in lower scaling-driven AMPAR upregulation, reduced imbalances in excitatory and inhibitory circuits, and lower susceptibility to ongoing damage.

## 1. Introduction

### 1.1. Synaptic scaling in Alzheimer's disease

Neuronal homeostatic synaptic scaling is a local feedback mechanism which senses levels of activity-dependent cytosolic calcium within the cell and adjusts neuronal firing activity accordingly. This is achieved by producing alterations in excitatory AMPA receptor accumulation in response to changes in firing activity occurring over hours to days (Turrigiano, 2008), leading to changes in the excitability of the neuron.

During learning, synaptic scaling plays an important role in balancing potentiation. By constantly shifting mean activation toward a target activity level, while maintaining the learned relative distribution of presynaptic weights, global levels of activity can be regulated (van Rossum et al., 2000; Rowan and Neymotin, 2013). During periods of hypoactivity (e.g., in degenerative disorders), synaptic scaling is also capable of raising the sensitivity of neurons via AMPA receptor upregulation, so that activity levels can be restored (Turrigiano, 2008).

Synaptic scaling has been postulated to play a key role in the progression of Alzheimer's disease (AD). The presence of Aβ has been shown to be a key mediator of calcium excitotoxicity in Alzheimer's disease (Demuro et al., 2010), and may explain the progression of the disease throughout functional neural networks (Small, 2008; Savioz et al., 2009). When neurons die and synapse efficacy is reduced in connection with β-amyloid (Aβ) and tau pathology in Alzheimer's disease, functionally connected neurons within the local network suffer a corresponding decrease in activation. As cells upregulate the number of AMPA receptors at synapses in order to compensate for this, the excitatory-inhibitory balance of the local network surrounding the cell is altered (Palop and Mucke, 2010). This can lead the cell to transition more easily to a high-excitation bursting state consisting of sustained firing or bursting at the neuron's saturation rate in synchrony with other connected cells (Fröhlich et al., 2008). This phenomenon has been observed in mouse models of AD, particularly in cells proximal to amyloid plaques, and correlates with the increased incidence of seizures in AD patients (Busche et al., 2008). These pathological states of high excitability, coupled with dysfunctions in Aβ-mediated calcium regulation, lead to greater influx of Ca^2+^ into the cell plasma, making the triggering of cell death (apoptosis) more likely. As this propensity to over-excitability increases in proportion to levels of compensatory synaptic scaling (Trasande and Ramirez, 2007), previously-healthy neurons which have scaled up to compensate for decreased local activation will also become destabilized and more susceptible to excitotoxicity, thus leading to a feed-forward cascade of apoptosis and neurodegeneration.

Small (2008) suggested targeting synaptic scaling mechanisms in AD as a potential method for preventing disease progression, but evidence suggests that this would have severe consequences on learning and regulation of synaptic activity (Turrigiano, 2011). It is likely that unconstrained potentiation during learning without compensatory downscaling would actually make neurons more likely to transition to damaging hyperactive states (Rowan and Neymotin, 2013). Instead, we propose targeting the root cause of this unbalancing homeostatically-induced up-scaling by counter-acting the decreased activation of remaining healthy neurons with long-term low-intensity prosthetic neurostimulation.

### 1.2. Neurostimulation to act on scaling mechanisms

Recent trials in probable Alzheimer's patients have reported sustained increases in neural activity following continuous (for 1 year) deep brain stimulation (DBS), with corresponding cognitive improvements (Smith et al., 2012). Other trials using transcranial magnetic stimulation (TMS) again showed improved cognitive performance after six months of daily applications of magnetic stimulation (Rabey et al., 2013). A review of trials using transcranial direct current stimulation (tDCS) also showed positive effects on cognitive performance, with effects persisting up to 4 weeks after stimulation (Hansen, 2012). These types of stimulation act by partially depolarizing the neural membrane, making it easier for natural synaptic events to trigger an action potential. However, as these trials are still in their early stages, the question of whether these treatments are merely masking the cognitive symptoms, or are actually halting the progression of the disease, remains to be answered.

In this work we use computational simulations to investigate the possible mechanisms by which applying mild sustained electrical neurostimulation to the cortex may be able to slow the progression of AD. Low-intensity informationless stimulation is able to restore activity and information transfer which was lost due to neighboring cell death and synapse dysfunction (Kerr et al., 2011), so we wondered whether such stimulation would also allowing global activity levels in the cortex to be maintained at a higher level after onset of Alzheimer's disease. Rather than neurons therefore having to scale up to account for lost activation, and thus destabilizing the excitatory-inhibitory balance and becoming susceptible to calcium excitotoxicity, the artificial stimulation causes the cells' scaling mechanism to suppress AMPAR upregulation, meaning levels of scaling are decreased and susceptibility to damage is reduced.

The effects of such neurostimulation on synaptic scaling and learning in Alzheimer's disease have not yet been investigated. In this work, we show how a 470-cell simulation of a neocortical sensory column incorporating homeostatic synaptic scaling is damaged by the progression of AD, and how electrostimulation can significantly slow the rate of this damage progression. We also investigate whether global stimulation is required, or whether more localized stimulation could also achieve the same results, and we show whether learning is adversely affected in the presence of such stimulation.

## 2. Materials and methods

### 2.1. Model description

**Figure 1:**
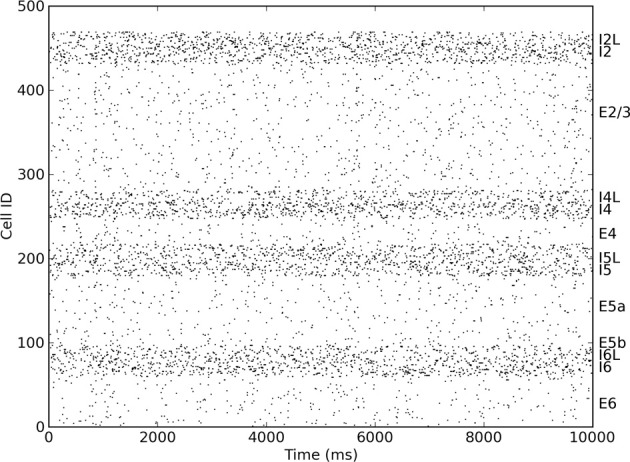
**Raster plot showing distinct firing rates of different excitatory and inhibitory layers within the cortical column**.

**Figure 2:**
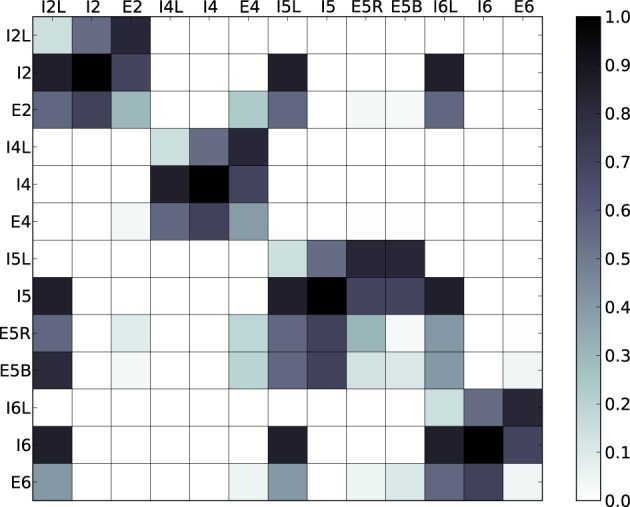
**Normalized probabilities of connectivity between and within the different cell populations in the network**. Vertical and horizontal axes represent cell types and color represents the normalized probability that a neuron from a given column will project to a neuron from a given row.

The spiking model used in this work is an extension of the neocortical model used in Neymotin et al. (2011a). It is based on the notion of a single cortical column (Binzegger et al., 2004; Lefort et al., 2009; Neymotin et al., 2011b) and consists of 470 neurons in three types (excitatory pyramidal cells E, fast-spiking inhibitory interneurons I, and low-threshold spiking inhibitory interneurons IL). Each of these neural types is distributed across the four neocortical layers in the model (layers 2/3, 4, 5, and 6), giving a total of 13 distinct neuronal populations (Figure 1). Numbers of cell in each type per layer are given in Table 1. The population sizes and wiring densities between and within layers were taken from multiple biologically-validated sources, as documented in Neymotin et al. (2011b). Figure 2 shows the relative density of connectivity between and within the different cell populations in the network.

**Table 1:** **Numbers of cell per type in each layer**.

**Layer**	I2L	I2	E2	I4L	I4	E4	I5L	I5	E5a	E5b	I6L	I6	E6
**Pop size**	13	25	150	14	20	30	13	25	65	17	13	25	60

*E2 = excitatory cell in layer 2/3, I2 = fast spiking interneuron in layer 2/3, I2L = low-threshold spiking interneuron in layer 2/3, etc. E5a and E5b are two subtypes of layer 5 pyramidal neurons*.

Each cell was modeled as a single-compartment integrate-and-fire neuron with fast inhibitory GABA_A_ receptors, fast excitatory AMPA receptors, and slow excitatory NMDA receptors, each producing a voltage step in the cell membrane potential *V*_*m*_ followed by a decay according to a synapse-specific delay constant. For speed, the cell state variables were only updated at the time of input events, with connectivity weights obtained using a *just-in-time* strategy to avoid the requirement to hold the entire of a massive weight matrix in memory, allowing fast and memory-efficient running even for large networks (Lytton et al., 2008). Biological behaviors such as adaptation, bursting, depolarization blockade, and voltage-sensitive NMDA conductance were simulated using a simple rule-based system (Lytton and Stewart, 2005, 2006).

Input events at synapses depotentiated the cell membrane voltage *V*_*m*_ by a specified reversal potential *E*_syn_, depending on the neurotransmitter type. The cell's default resting membrane potential *V*_RMP_ was given as a baseline, from which *V*_*m*_ was updated. If *V*_*m*_ exceeded the spiking threshold *V*_θ_ following a synaptic event, the cell emitted a spike before entering a refractory period of τ_refrac_ ms. The cell could not fire during the refractory period. This was used to set an upper limit on firing frequency. Resting potential, threshold and refractory periods for cells in each population type are given in Table 2.

**Table 2:** **Cell resting potential, threshold, and refractory period, for each cell population**.

**Cell type**	*****V***_**RMP**_ (mV)**	*****V***_θ_ (mV)**	**τ_**refrac**_ (ms)**
I2L	−65	−47	10
I2	−63	−40	10
E2	−65	−40	50
I4L	−65	−47	10
I4	−63	−40	10
E4	−65	−40	50
I5L	−65	−47	10
I5	−63	−40	10
E5a	−65	−40	50
E5b	−65	−40	50
I6L	−65	−47	10
I6	−63	−40	10
E6	−65	−40	50

Each AMPA, NMDA, and GABA_A_ synapse had its own voltage state *V*_*syn*_, which was added to the cell's membrane potential *V*_*m*_. After each synaptic event, *V*_*syn*_ decayed exponentially with time constant τ_syn_. GABA_A_ events were simulated in two different ways: directly at the cell soma with a fast decay time-constant, and at the dendrite with a slower time-constant, giving two separate *V*_*syn*_ states. Synaptic events had a small delay before updating *V*_*m*_, chosen uniformly from a distribution τ_delay_.

Network activity was driven by spikes from neighboring cells, as well as subthreshold external inputs representing activation from other areas of the brain, necessary to prevent intrinsic activity from dying out. Spikes generated by a Poisson process were provided at each synapse of each cell, maintaining average input frequencies drawn uniformly from a distribution *f*_ext_. Reversal potential, *V*_*syn*_ decay constant, synaptic delay and external input frequency for each synapse type are given in Table 3. In some simulations, an additional low-amplitude (but super-threshold) training signal was applied to layer 4 excitatory neurons (E4), representing sensory input to the neocortex.

**Table 3:** **Reversal potential, *V*_*syn*_ decay constant, synaptic delay and external input frequency for each synapse type**.

**Synapse type**	*****E***_**syn**_ (mV)**	**τ_**syn**_**	**τ_**delay**_ (ms)**	*****f***_**ext**_ (Hz)**
AMPA	65	20	3–5	240–360
NMDA	90	300	3–5	40–60
GABA_A_ (soma)	−15	10	3–5	100–150
GABA_A_ (dendrite)	−15	20	1.8–2.2	100–150

Further details of the cell model, including activity blockade, relative refractory periods, and after-hyperpolarization rules, in addition to wiring densities, are given by Neymotin et al. (2011b) and Neymotin et al. (2011a).

### 2.2. Synaptic scaling

The model was extended to implement synaptic scaling at E cell AMPA synapses by multiplying each cell *i*'s postsynaptic input by a scale factor *c*_*i*_, representing the multiplicative accumulation of AMPA receptors at synapses. Changes in the scale factor were calculated following the formula of van Rossum et al. (2000), with *a*_*i*_(*t*) as the cell's instantaneous firing rate at time *t, a*^*goal*^_*i*_ as the target firing rate, β as the scaling strength, γ as the “integral controller” weight, and $\frac{dc_{i}(t)}{dt}$ as the rate of change of the synaptic weight:

(1) $$\frac{dc_{i}(t)}{dt}=\beta c_{i}(t)[a_{i}^{\;goal}-a_{i}(t)]+\gamma c_{i}(t)\int_{0}^{t}dt[a_{i}^{\;goal}-a_{i}(t)]$$

in which the first term modifies the synaptic weight according to the current disparity between *a*^*goal*^_*i*_ and *a*_*i*_(*t*), and the second term allows historical under- or over-shoots of activity to pull on the weight updates more strongly, the longer they continue.

Scaling was applied inversely at GABA_A_ synapses by multiplying postsynaptic input by $\frac{1}{c_{i}}$ to enable the scaling of excitatory and inhibitory synapses in opposite directions. This allows mimicking of the effect of activity-dependent global growth factors such as BDNF, which is known to enhance activity in inhibitory circuits as excitatory output increases (Rutherford et al., 1998; Turrigiano, 2008, 2011; Chandler and Grossberg, 2012). This produces a different result to merely varying the firing threshold of the neuron: with homeostatic scaling only at excitatory synapses, an increase in E cell excitation would lead to a corresponding increase in excitation of the inhibitory cells. This would then suppress E activity once more, and effectively counteract the scaling effect. But by scaling down the inhibitory synapses onto E cells at the same time as scaling up the excitatory synapses, the increased I cell activity (resulting from increased E cell activity) will be weighted lower by the E cells, thereby maintaining the increase in E cell activity caused by scaling, whilst also maintaining I cell firing rates.

The following parameter values were used for synaptic scaling: strength β = 4.0 × 10^−8^/ms/Hz; integral controller weight γ = 2.0 × 10^−10^/ms^2^/Hz; activity sensor time constant τ_*a*_ = 100 × 10^3^ ms. Individual cell scale factors were bounded to 100, to prevent biologically-unrealistic levels of scaling (representing AMPA receptor accumulation) increasing to infinity.

Instantaneous firing rates for each cell *i* were sensed using van Rossum's slow-varying sensor *a*_*i*_(*t*), which increased monotonically with spikes at times *t*_*x*_ (given by the Dirac delta function δ), and decayed otherwise (van Rossum et al., 2000):

(2) $$\tau_{a}\frac{da_{i}(t)}{dt}=-a_{i}(t)+\sum_{x}\delta (t-t_{x})$$

The sensor decays exponentially as it is updated at each non-firing timestep. However, the use of event-driven just-in-time synapses in the model (Lytton et al., 2008; Neymotin et al., 2011a) meant that cell states were only updated at each spike event rather than at every timestep, so inter-spike decay of the activity sensor could only be calculated periodically. The activity sensor was therefore modified to cope with periodic-timestep updates. Here, the first term decays the sensor according to the time since the last spike *t* − *t′*, and the second term increments it for the new spike, with both terms updated concurrently on the occurrence of a spike at time *t*:

(3) $$a_{i}(t)=a_{i}(t^{\prime })e^{-\frac{1}{\tau_{a}}(t\;-\;t^{\prime })}+\frac{1-a_{i}(t^{\prime })}{\tau_{a}}$$

**Figure 3:**
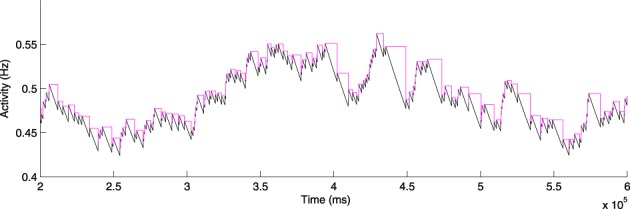
**Activity sensor updating at every simulation timestep (Equation 2; black) and at every spike for activity-driven just-in-time synapses (Equation 3; magenta)**.

Figure 3 shows the activity sensor values of a simulated uniform-random spiking neuron operating under the constant-timestep update policy (2), and the equivalent activity values under the periodic-update policy (3). The activity rises identically in both cases when spikes occur, but the periodic sensor does not decay until the next spike event occurs, giving the step-like appearance. The values at the spike times are therefore correct down to round-off error at the spike times.

To set the target firing rate *a*^*goal*^_*i*_ for each neuron, and thus also the global total activity *A*^*goal*^, there are two options. The first would be to provide an arbitrary firing rate target (say, 0.5 Hz) for each cell, but this would be likely to fundamentally affect the network dynamics by over-riding the natural firing rate of each neuron. Instead, the intrinsic dynamics of the network were used to provide information on the natural firing rates of neurons, which (once stabilized over time) were then taken as the target activity rates. Initially, with synaptic scaling off, activity sensors began at *a*_*i*_(*t*_0_) = 0 Hz. They were then adjusted over 1600 s of simulated time based on the actual activity of the cells, according to Equation 3. Synaptic scaling was then switched on, with the value of the activity sensor at that time used as the ongoing target firing rate:

(4) $$a_{i}^{\;goal}=a_{i}(t=1600\;s)$$

The global activity target (section 2.2.1) was then set as

(5) $$A^{\;goal}=\sum_{i}a_{i}^{\;goal}$$

#### 2.2.1. Global synaptic scaling driven by neurotrophic factors

Small (2008) predicts that not only should cells homeostatically scale back up to their target activity in order to compensate for lost activation, but that the network as a whole should also scale up its activity in response to damage, in order to maintain a global absolute firing rate and therefore maintain information processing throughput. Individual cells are therefore required to alter their activity targets, so they can scale up beyond their original “design” firing rate to compensate for the global loss of activity. Such a mechanism would require scaling to be governed by a global activity signal in addition to the local signal generated by each cell's activity sensor. This global signal takes the form of a combination of a reduction in the release of the activity-dependent neurotrophin BDNF, and an increase in the release of the growth factor TNF-α from neighboring glial (support) cells (Turrigiano, 2008).

Such neurotrophic scaling was incorporated into the simulations by calculating a global scaling signal *C* as a proportion of global activity *A*, and comparing this to the baseline target global activity *A*^*goal*^:

(6) $$A(t)=\sum_{i}a_{i}(t)$$

(7) $$C(t)=\frac{A^{\;goal}}{A(t)}$$

Synaptic scaling for each cell (Equation 1) was then adjusted to include the global scaling signal *C* as a multiplier of the usual target activity *a*^*goal*^_*i*_:

(8) $$\frac{dc_{i}(t)}{dt}=\beta c_{i}(t)[C(t)\cdot a_{i}^{\;goal}-a_{i}(t)]+\gamma c_{i}(t)\int_{0}^{t}dt[C(t)\cdot a_{i}^{\;goal}-a_{i}(t)]$$

This had the effect of allowing the target activity level of a cell to be adjusted dynamically up and down in response to global scaling signals, as hypothesized by Turrigiano (2008), but with the scaling mechanism still applied locally at each cell based on changes in its postsynaptic input.

### 2.3. Cell death

Small (2008) hypothesized that, as cells are driven to over-activity beyond their target firing rates, the subsequent influx of activity-dependent calcium ions – exacerbated by the β-amyloid pathology present in AD (Demuro et al., 2010) – triggers an apoptopic cell-death mechanism. The cell is programmed to die safely and cleanly in order to prevent more damaging *necrosis*, in which the cell dies, its membrane breaks down, and parts of the dead cell are leaked into the intracellular space, provoking further damage. Such over-activity may be caused by chronic over-activation following significant compensatory scaling, or by activity peaks caused by synchronized network-wide bursting, or even by cells being over-activated by strongly-innervating neurons to which they are connected.

In simulations with excitotoxicity, cell death for inhibitory and excitatory cells was modeled by calculating, at the time of each synaptic event, the ratio of the error between the cell's current activity value and its target activity, normalized by its target activity:

(9) $$a_{i}^{err}=\frac{a_{i}(t)-a_{i}^{\;goal}}{a_{i}^{\;goal}}$$

If *a*^*err*^_*i*_ was greater than a threshold value, set in simulations to 1.5 times the goal activity, then a probability of cell death *P*^*death*^_*i*_ was calculated, equal to the activity error multiplied by a deletion rate constant τ_*del*_. *P*^*death*^_*i*_ was also multiplied by the scaling factor value of the cell, to model the effects of scaling-dependent excitotoxicity, and by the time since the last synaptic update at time*t*′_*i*_:

(10) $$P_{i}^{\;death}(t)=\tau_{del}\cdot a_{i}^{err}c_{i}(t-{t^{\prime }}_{i})$$

To prevent external inputs from dominating the dynamics as internal connectivity was reduced, the external input weights were globally reduced in proportion to the number of remaining cells *N*(*t*) in the network, subject to a scaledown rate constant τ_*ext*_:

(11) $$w_{ext}(t)=1-\frac{N_{0}-N(t)}{N_{0}}*\tau_{ext}$$

In order to start the feed-forward process of cell death, it was necessary to provoke compensatory up-scaling throughout the network. During normal running, the activity levels, *a*_*i*_, and subsequent scaling multipliers, *c*_*i*_, of each cell fluctuate slightly. At the time of disease onset, the 15 cells with the highest *c*_*i*_ were deleted manually, using the assumption that these cells would be most susceptible to scaling-related pathology. This caused a reduction in global activity, causing the remaining cells to scale up above baseline levels, and enabling the onset of the damage cascade via Aβ-mediated cell death.

The deletion process was initiated after 17,600 s of baseline activity (with scaling initiated after the first 1600 s), to allow synaptic scaling sufficient time to stabilize, and to identify the cells which would scale up the most. τ_*del*_ took the value 1 × 10^−4^, giving a level of deletion of approximately 78 ± 10% of cells after 2 days of simulated time. Altering τ_*del*_ was found to have only linear effects on the global deletion rate.

### 2.4. Electrostimulation

Electrostimulation was implemented in the form of a regular Poisson-generated pulse applied to all of the excitatory cells in the specified population(s). The frequency of stimulation and unitless weight multiplier [broadly analogous to the stimulation strength in tDCS or TMS applications, typically around 2 mA (Hansen, 2012)], could be varied. The mechanism of action of tDCS and TMS is to partially depolarize the cell membrane, thus essentially lowering the activity threshold required to fire an action potential. Ideally, this would be implemented in the model as a direct modifier of the membrane potential, but due to limitations in the simulation environment it was necessary to apply the stimulation indirectly via AMPA synapse injection. As AMPA receptors were subject to homeostatic scaling, this would also have led to scaling of the electrostimulation. However, stimulation should be scaling-independent as it operates directly on the cell membrane. Therefore, it was necessary to “de-scale” the electrostimulation weight at each cell's AMPA synapses by multiplying by $\frac{1}{c_{i}}$.

**Figure 4:**
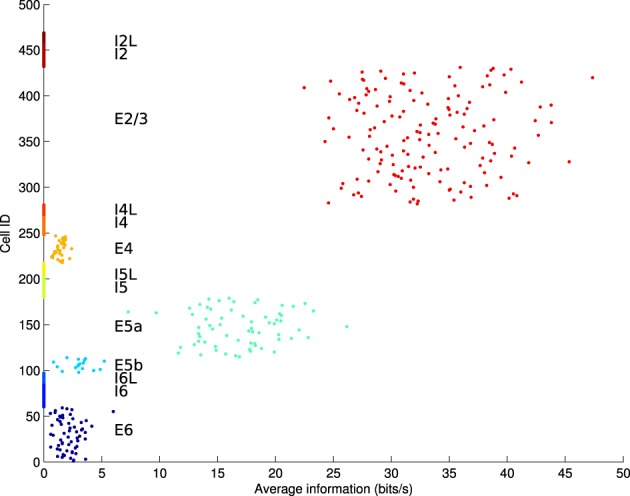
**Average information contribution (in bits/s) per cell over 20 runs, arranged by cell population**. Only excitatory cell populations were tested. E2/3 is the layer with the highest information contribution, followed by E5a.

In some simulations, electrostimulation was applied to only a single layer of the model, in order to differentate between localized and global stimulation (e.g., modeling a local neuroprosthesis vs. global tDCS). To identify the most appropriate layer of the model for stimulation, mutual information measures were obtained for each individual excitatory cell between that cell and the rest of the network. This was achieved using the Fourier spike-train analysis method of Yu et al. (2010); Crumiller et al. (2011). The network was driven by high-intensity stimulation to elicit spike trains which displayed causality between each cell and the remainder of the network. The results of this information-probing, averaged over 20 runs, showed layer E2/3 cells to have the highest contribution of information to other cells within the network (Figure 4). Additionally, E2/3 is one of the thickest layers of the cortex, making it physically easier to target with a prosthetic implant.

### 2.5. Computational setup

An activity sensor time constant τ_*a*_ of 100 s (van Rossum et al., 2000) leads to a simulation timescale of several hours for synaptic scaling: far closer to the expected biological timescale than previous studies (Fröhlich et al., 2008; Turrigiano, 2008; Chandler and Grossberg, 2012). To achieve this length of simulation, the model was extended to allow periodic flushing of all spike data to disk, enabling very long runs (unlimited except for available disk space). A typical simulation of 44 h ran approximately 1–2 times faster than real time and produced around 2–4 GB of spike data per run. Experiments were run on the BlueBEAR cluster at the University of Birmingham, UK. The model was implemented in NEURON 7.2 (Carnevale and Hines, 2006) for Linux, and is available on ModelDB (https://senselab.med.yale.edu/modeldb).

### 2.6. Information transfer

To discover whether electrostimulation negatively affects information processing within the network at baseline, normalized transfer entropy (nTE) measures were obtained at baseline with and without stimulation.

Standard transfer entropy (Equation 12) reveals the direction and quantity of information transferred between two processes *X*1 and *X*2:

(12) $$TE_{X1\to X2}=H\left(X2_{f}|X2_{p}\right)-H\left(X2_{f}|X2_{p},X1_{p}\right)$$

in which the subscripts *f* and *p* refer to the *future* and *past* outcomes of *X*1 and *X*2. In our experiments, *X*1 and *X*2 were represented by multi-unit activity vectors (bin size 10 ms) for each neuronal population in turn, so that each population's total information transfer to all other populations could be determined.

In the case that both processes *X*1 and *X*2 are in fact driven by a third hidden process which determines the time course of *X*1 and *X*2, the standard transfer entropy calculation makes an invalid assumption regarding the contribution of *X*1–*X*2. To avoid this situation, normalized transfer entropy (nTE) was developed by Gourévitch and Eggermont (2007). The nTE measure eliminates bias by shuffling the representation of the process *X*1 and subtracting its average over many shuffles from the estimate of transfer entropy, finally normalizing the value by the entropy of *H*(*X*2_*f*_ | *X*2_*p*_):

(13) $$nTE_{X1\to X2}=\frac{TE_{X1\to X2}-\langle TE_{X1_{s}\to X2}\rangle }{H\left(X2_{f}|X2_{p}\right)}$$

where *X*1_*s*_ denotes the shuffled form of *X*1.

In simulations measuring information transfer, the network was driven with sensory input inserted via layer E4. This was a 90-s local field potential recording obtained during a previous experiment (Kerr et al., 2011) from a single electrode inserted into the VPL nucleus of the thalamus of a female Long-Evans rat, and filtered using a 3rd-order Butterworth bandpass filter with cutoffs at 5 and 200 Hz.

## 3. Results

The simulation was run 50 times for each experiment, with each run taking different random seeds determining initial wiring layout, external Poisson noise inputs, cell placement, and internal synaptic strengths on each run.

**Figure 5:**
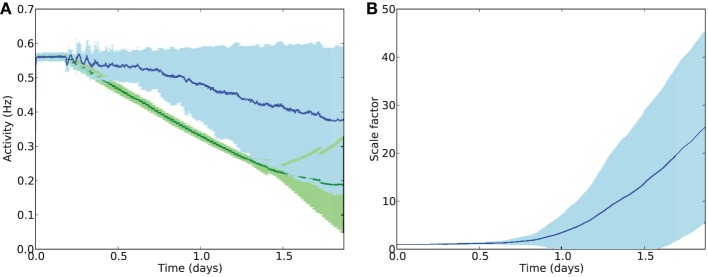
**(A)** Mean firing rates across all E cells during deletion with (blue) and without (green) compensatory scaling. Error patches show standard error of the mean E cell activity over 50 runs. It should be noted that cell death is greater in the case of AD-like deletion with compensatory scaling (78 % of E cells on average) compared to uniform deletion (64 % of E cells deleted); average firing rates are better maintained with synaptic scaling despite greater damage. **(B)** Scaling factors during AD-like deletion increase rapidly after the onset of damage.

The deletion rate constant was set to τ_*del*_ = 0.0001 which, under normal conditions, resulted in a smooth progression to near-complete cell death within the timescale of one run of the 44-h simulation (Figure 5). Clearly this is far faster than biological cell atrophy in AD, but such a deletion rate was required to be able to show the effects of disease progression within the available time. It should be noted that the speed of deletion is linearly proportional to τ_*del*_, and that the following experiments only compare relative speeds of progression with/without stimulation, not absolute speeds. Unless otherwise stated, the following parameters values were used: synaptic scaling strength β = 4.0 × 10^−8^/ms/Hz; integral controller weight γ = 2.0 × 10^−10^/ms^2^/Hz; activity sensor time constant τ = 100 × 10^3^ ms; electrostimulation weight = 2.0, electrostimulation frequency = 3 Hz, external input scaledown constant *T*_*ext*_ = 0.25.

### 3.1. Synaptic scaling restores lost activity

To provide a baseline against which to compare further experiments, deletion was performed by selecting a fixed number of cells at random at each deletion timestep. Three inhibitory or excitatory cells were picked at uniform random every 1600 s, over 100 rounds of deletion during the simulation, giving a total of 300 deleted cells (around 64% of the network) by the end of the simulation (Figure 5). Next, scaling was added to the network and random cell death was replaced with scaling-driven progression of pathology, as detailed in the Methods. Synaptic scaling was used to bring each excitatory cell back toward its target firing rate.

Despite greater cell death during AD-like deletion (on average around 78% of cells by the end of the simulation), the compensatory synaptic scaling maintained average firing rates for much longer than in the absence of scaling. Analysis of the scaling factor values *c*_*i*_ across all E cells showed a dramatic increase in multiplicative compensatory scaling during the deletion process, averaging nearly 30 times the baseline value by the end of the simulation period.

### 3.2. Global neurostimulation slows progression of damage

**Figure 6:**
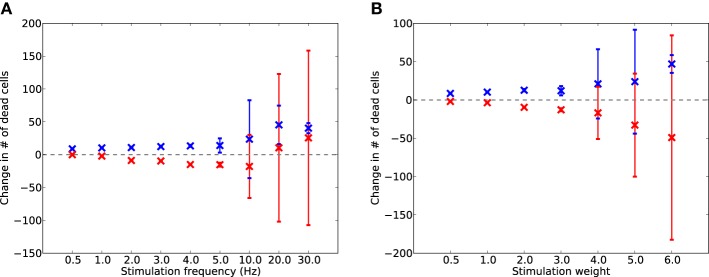
**Change in dead E (red) and I (blue) cells after 2 days of AD-like scaling-driven damage, compared to cell death levels without stimulation, across varying frequencies (A) and weights (B) of electrostimulation**. Error bars show standard deviation over 50 runs.

To test whether electrostimulation is capable of replacing lost activity during damage, suppressing excessive synaptic scaling, and therefore slowing the progression of damage, the simulation was run for a further 2 days with synaptic scaling enabled. Continuous stimulation was provided after 17,600 s at various frequencies from 0.5 to 30 Hz, and at various unitless intensities from 0.5 to 6.0. The levels of cell death for each run (of 50, each with different random initial seeds) were compared to the levels of cell death in the equivalent run without stimulation; the resulting differences are shown in Figure 6.

Electrostimulation at low frequencies (1–10 Hz) reduced the rate of cell death in E cells (Figure 6A) by up to 5%. However, at 10 Hz and above, there was an increase in the death rate of I cells. This was due to the I cells having to provide extra inhibitory activity to balance the increased drive from the electrostimulation, suggesting that stimulation at these higher frequencies is detrimental to the network during disease progression. A similar effect was seen with stimulation weight (Figure 6B), with increasing weight successfully slowing the rate of E cell death by up to 18% at all intensities tested, but actually contributing to faster I cell death at weights above 4.0.

**Figure 7:**
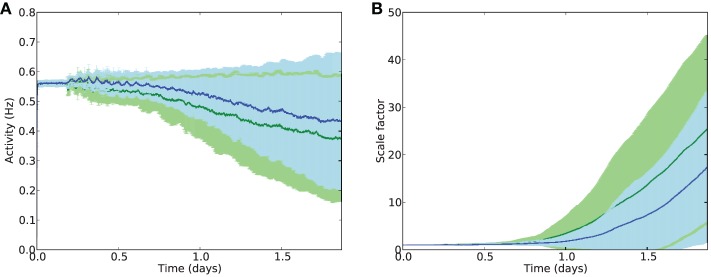
**Average activity (A) and scaling factors (B) of E cells during AD-like scaling-driven deletion without (green) and with (blue) electrostimulation (4 Hz, strength 2.0)**.

Examining the effects of electrostimulation on excitatory firing activity and rates of compensatory synaptic scaling shows that the stimulation is capable of further maintaining E cell activity in combination with synaptic scaling (Figure 7A), with average E firing rates after 2 days of damage at around 0.43 Hz with stimulation, compared to 0.38 Hz without stimulation. Similarly, synaptic scaling is shown to be suppressed by electrostimulation (Figure 7B).

These results suggest that electrostimulation could lead to a reduction in the rate of cell death in synaptic scaling-driven AD-like atrophy situations, through providing additional compensatory activity and reducing the need for unbalancing compensatory synaptic scaling. However, the choice of frequency and intensity of stimulation is important, as stimulation at too high a frequency or intensity may instead cause further damage, particularly through over-stimulation of the inhibitory system.

### 3.3. Localized stimulation via neuroprosthesis

Global stimulation in a clinical setting (e.g., via tDCS) requires repeated visits to a treatment center, and such high-energy stimulation is neither targeted, nor currently certified as safe for more than 20 min of stimulation per 48 h (Utz et al., 2010). High-intensity and/or long-duration stimulation has been shown to induce brain lesions in rats (Liebetanz et al., 2009). A more practical solution for long-term low-intensity treatment for AD may be to induce continual low-intensity stimulation via an implant, such as is currently achieved with Parkinson's disease patients. To investigate whether non-global stimulation could have similar therapeutic effects to global stimulation, we inserted a simulated prosthetic stimulation into the cell population E2/3, as this layer is known to be a driver of neural activity throughout the rest of the microcircuit (Weiler et al., 2008; Neymotin et al., 2011b), and information-contribution measures show that it is the most influential layer on overall network activity (Figure 4).

**Figure 8:**
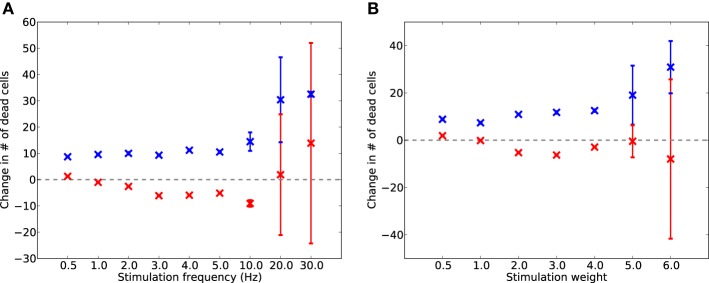
**Local electrostimulation of layer E2/3: change in dead E (red) and I (blue) cells after 2 days of AD-like scaling-driven damage, compared to cell death levels without stimulation, across varying frequencies (A) and weights (B) of stimulation (50 experimental runs)**.

**Figure 9:**
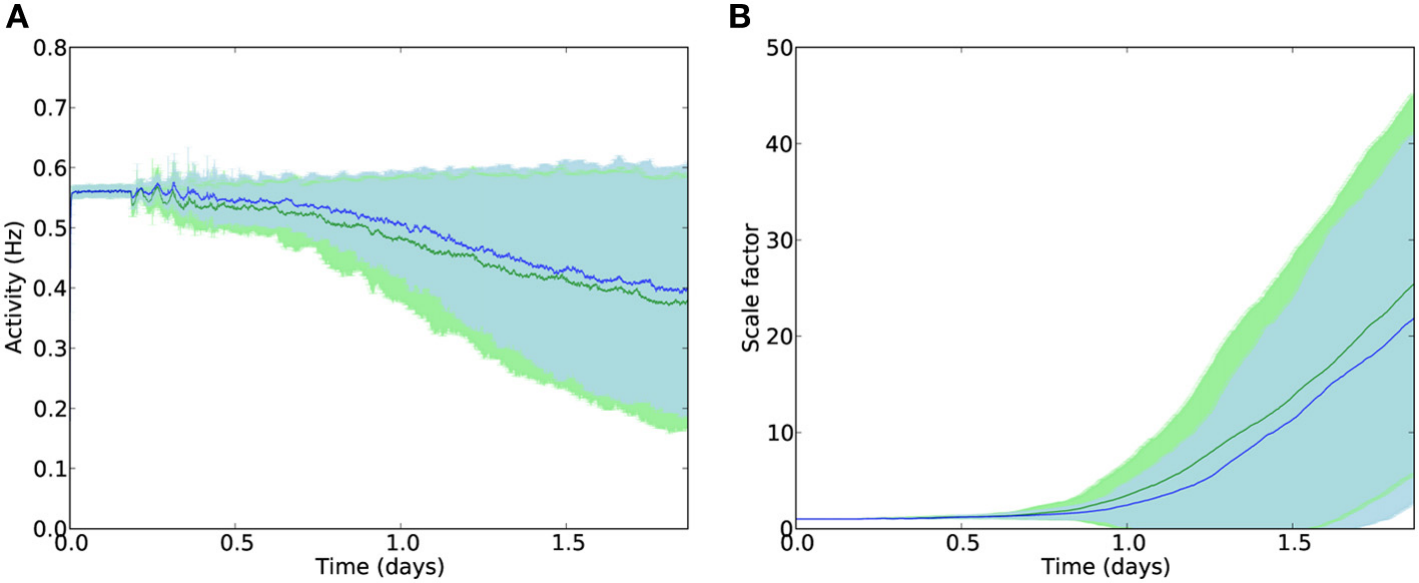
**Average activity (A) and scaling factors (B) of E cells during AD-like scaling-driven deletion without (green) and with (blue) localized E2/3 electrostimulation (4 Hz, strength 2.0)**.

The results in Figure 8 indicate that, although the localized stimulation reduced E cell death up to 10 Hz (weight = 2.0) or up to weight 4.0 (frequency = 4 Hz) by up to 3%, the effect on cell death is reduced in comparison to global stimulation (Figure 9). Similarly, the effect on mean firing rates and reduction of scaling is present, but smaller than in the whole-brain stimulation case (Figure 9B). This is not surprising in itself, but it shows that local stimulation could still make a usable alternative to whole-brain stimulation.

### 3.4. Effects of stimulation on information transfer

To discover whether electrostimulation negatively affects information processing within the network at baseline, normalized transfer entropy (nTE) measures were obtained at baseline with and without neuro-stimulation (see Methods).

**Figure 10:**
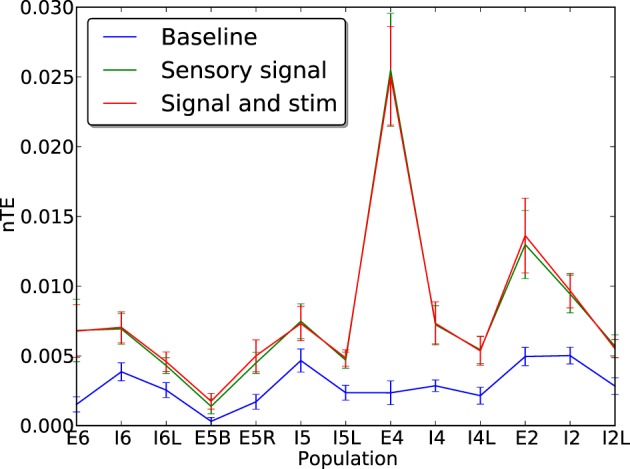
**Normalized transfer entropy (nTE) measures for each cell population with (green) and without (blue) replay of a recorded rat LFP signal inserted via layer E4**. The sensory signal reveals the true information transfer rate of the network in the presence of an informative signal. Addition of informationless electrostimulation on top of the sensory signal does not affect the ability of the network to transfer information. Error bars show standard error of the mean over 50 runs.

**Figure 11:**
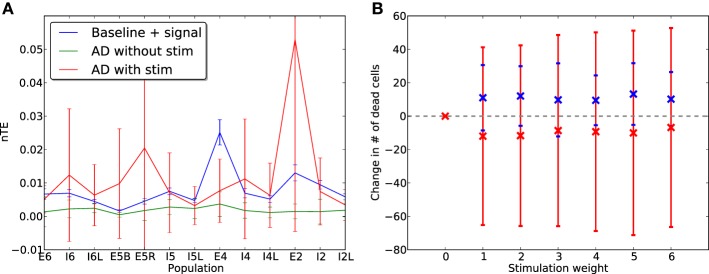
**(A)** Normalized transfer entropy (nTE) measures for each cell population at baseline with the rat LFP signal inserted via layer E4, before damage (blue), and after one day of damage with (red) and without (green) therapeutic electrostimulation (4 Hz, weight 2.0). **(B)** Average change in cell death for E (red) and I (blue) cells after 2 days of AD-like damage with electrostimulation and E4 sensory signal, compared to baseline (50 runs).

In these simulations, the network was driven with sensory input inserted via layer E4. This was a 90-s local field potential recording obtained during a previous experiment (Kerr et al., 2011) from a single electrode inserted into the VPL nucleus of the thalamus of a female Long-Evans rat. The inclusion of this sensory signal raised the baseline firing rates and increased the effectiveness of communication between cells, so that information transfer could become visible compared to baseline. Addition of neuroprosthetic stimulation did not interfere with the flow of information from the E4 sensory signal, with the nTE measures remaining largely unchanged in the presence of stimulation (Figure 10). In addition, to determine whether stimulation in the case of AD-like damage is capable of maintaining information processing capabilities, nTE measures were obtained at a point mid-way through the damage cascade (1 day), in situations with and without stimulation (4 Hz, weight 2.0; Figure 11A). The results show a clear improvement in the information transfer within the network with prosthetic stimulation, in comparison to the network without stimulation in which the nTE values were nearly 0. With stimulation, the nTE values during damage were close to the pre-damage baseline nTE values, albeit with greater variance.

Although the mean information transfer in the damaged network with stimulation remained comparable to baseline, there was a notable shift of the peak nTE values from the E4 population toward the E2/3 and E5a populations. The resulting distribution of information contribution across populations mirrors that seen in Figure 4, which was obtained under conditions of high intensity stimulation. This suggests that under a regime of stimulation, the network became more and more driven by internal signals rather than the external E4 sensory signal, which could be a possible warning sign for the generation of stereotyped activation patterns seen in autism (Qiu et al., 2011; Shepherd, 2013).

After 2 days of simulated damage, the change in proportion of dead E and I cells was calculated (Figure 11B). As before, increasing the intensity of stimulation led to a decrease in E cell death rates of up to 5%, but the negative effect on I cells was more pronounced even at low stimulation intensities when the network was driven with a sensory signal via E4.

## 4. Discussion

In atrophic disorders such as Alzheimer's disease, cell death leads to compensatory up-scaling of excitatory AMPA receptors in remaining cells. This is hypothesized to induce instabilities as small, localized fluctuations in cell activity become magnified and lead to hyperactivity of nearby healthy neurons. In the presence of Aβ (which mediates calcium excitotoxicity in Alzheimer's disease), these transient hyperactive events cause excitotoxic cell death in remaining healthy cells and send the network into a feed-forward cascade of damage.

We have described a mechanism by which therapeutic low intensity, low frequency electrostimulation could act on homeostatic synaptic scaling mechanisms to reduce the pathological effect of excessive compensatory scaling in these diseases. The achieved reductions in E cell death are modest both in the whole-brain (e.g., tDCS) and the safer localized stimulation (e.g., neuroprosthesis) case: between 2 and 5% depending on the type and location of stimulation. However, this reduction in cell death, coupled with the general restoration of activity by the electrostimulation, is sufficient to maintain average firing rates of E cells better than with synaptic scaling alone, and crucially is also able to maintain average information transfer rates between cell populations compared to the baseline disease case.

It should be noted that the exact frequencies and intensities of stimulation chosen in this work are specific to the present model, and cannot be directly translated into a clinical setting. Stimulation in a different model, or in a clinical setting, would necessitate experimentation to find appropriate values. Our results show the changes in cell death for *relative* changes in stimulation frequency and intensity above the baseline AD state.

Such stimulation could be applied via either localized prosthetic stimulation, or by global whole-brain stimulation (e.g., tDCS). Localized prosthetic stimulation has clear benefits in portability and directness of action compared to whole-brain stimulation, which currently requires tightly-controlled sessions of stimulation in a clinical setting. Our model has shown that such localized stimulation in layer E2/3 can still have positive, albeit reduced, effects on cell death rates, although our model has not looked at the ability of such stimulation to act across multiple columns of the cortex, so it is possible that multiple prostheses would need to be used across the brain. Prosthetic stimulation has a good track record in treatment of Parkinson's disease (Modolo and Beuter, 2009), albeit at high frequencies which our model suggests will actually enhance damage in degenerative disorders, so the development of a low-frequency neuroprosthetic device for treatment of Alzheimer's disease already has precedent.

Clearly, testing the long-term outcomes of such stimulation would be difficult, as such a clinical trial would take many years to complete. Nevertheless, initial 1-year studies have indicated positive responses in suspected AD patients to gentle electrostimulation (Hansen, 2012; Smith et al., 2012; Rabey et al., 2013). The proposed mechanism of action of electrostimulation on suppressing excessive synaptic scaling could be tested *in vitro* by applying non-fatal signal-blocking toxins to a neuronal cell culture. The resulting increases in synaptic scaling as the cells compensate for the reduction in activity, measured as changes in AMPA-mediated synaptic currents, could be obtained according to the method of Turrigiano et al. (1998). Repeating the experiment with a second cell culture, but in the presence of low-intensity electrostimulation, should yield smaller changes in the upregulation of AMPA receptor concentrations. It would also be interesting to note whether firing rates are more stable in this case than in the case without electrostimulation. Finally, it has already been shown by Yu et al. (2010) and Crumiller et al. (2011) that their Fourier information measure can be applied to networks of biological neurons. Information measures for each cell in the culture could be obtained using this method, and compared with the predictions made in this paper for the distribution of information transfer with/without electrostimulation and before/after damage.

One interesting observation from our model is of a shift of the network dynamics from being externally-driven via E4 input, to becoming more strongly internally-driven (primarily via E2/3 and E5a) during long-term stimulation, suggesting a possible risk factor for stereotyped activity associated with disease (Qiu et al., 2011; Shepherd, 2013), resulting from such stimulation.

The model does not currently include the effects of tau pathology, which is expected to operate more on synapses than on whole cells. However, we expect the results to be largely similar, as synaptic dysfunction leads to lower activation of cells, with subsequent compensatory scaling and greater excitability still leading to excitotoxicity.

### 4.1. Justification for our approach

Due to the high computational costs of running multiple long (2-day) simulations, the model used in this work consisted of only a single cortical column. The model exhibits recurrent connectivity within layers and feed-forward connectivity between layers based on anatomical data. Previous work with the same model (but using much shorter simulation runtimes) has shown how, in a multi-columnular architecture which includes additional feed-forward lateral connectivity between columns, excitation spreads first within, and then across, columns (Neymotin et al., 2011b). Since our hypothesis is based on the principle that reduced excitation in the presence of AD pathology leads to destabilizing compensatory upscaling in the remaining cells, it follows that reduced excitation within one column would lead to reduced intra-columnular lateral excitation as well, and therefore the same spread of pathology not only within, but between columns.

We also note that AD involves a complex process that occurs at multiple temporal (from seconds to years) and spatial (molecular, cellular, network, brain area, behavior) scales (Lytton et al., 2014). Due to the complexity of the different subsystems involved, our model is only a coarse approximation that necessarily leaves out many of the details and interactions that occur *in vivo*. However, within its own scale, our model offers several experimentally-testable predictions, which may suggest further research to be performed *in vitro* or *in vivo*.

The Hodgkin-Huxley (HH) model provides a rich formalism for describing electrical dynamics in neurons contributing to action potential generation. The HH formalism also allows specification of multiple classes of ion channels, some of which admit calcium. Calcium is an ubiquitous second messenger, involved in regulating neuronal learning, plasticity, and which also contributes to excitotoxicity and ischemia, which are associated with different disease processes. Although electrical and calcium dynamics are crucial for fully understanding how AD develops in neurons, adding these dynamics into our model would drastically increase computational costs. In addition, some of the details are left out in order to simplify the model and allow us to focus on homeostatic synaptic scaling, which only requires spiking neurons. By using our simplified model and deriving predictions from it, more detailed models could be developed with HH neurons, and using the same rules of synaptic scaling.

A strength of our approach is that we can look at much longer simulation runs (due to lower computational costs) as well as more parameter variations. Therefore, for the purposes of our investigation, we have investigated network effects that are presumed to work via synaptic scaling. Networks of the neuron models we are using display similar dynamics with networks of HH neurons (Neymotin et al., 2011b; Rowan and Neymotin, 2013). Since the effects we are interested in can be simulated with a less detailed neuron model without connecting to intracellular calcium dynamics, we believe our simplifications are justified.

### Conflict of interest statement

The authors declare that the research was conducted in the absence of any commercial or financial relationships that could be construed as a potential conflict of interest.
